# Point Mutations as Main Resistance Mechanism Together With P450-Based Metabolism Confer Broad Resistance to Different ALS-Inhibiting Herbicides in *Glebionis coronaria* From Tunisia

**DOI:** 10.3389/fpls.2021.626702

**Published:** 2021-04-01

**Authors:** Zeineb Hada, Yosra Menchari, Antonia M. Rojano-Delgado, Joel Torra, Julio Menéndez, Candelario Palma-Bautista, Rafael de Prado, Thouraya Souissi

**Affiliations:** ^1^Department of Plant Health and Environment, National Institute of Agronomy of Tunisia, University of Carthage, Tunis, Tunisia; ^2^Laboratory of Bioagressor and Integrated Management in Agriculture (LR14AGR02), National Institute of Agronomy of Tunisia, University of Carthage, Tunis, Tunisia; ^3^Higher Institute of Biotechnology of Beja, University of Jendouba, Jendouba, Tunisia; ^4^Department of Agricultural Chemistry and Soil Science, University of Córdoba, Córdoba, Spain; ^5^Department d’Hortofructicultura, Botànica i Jardineria, AGROTECNIO-CERCA Center, Universitat de Lleida, Lleida, Spain; ^6^Departamento de Ciencias Agroforestales, Escuela Politécnica Superior, Campus Universitario de La Rábida, Huelva, Spain

**Keywords:** ALS enzymatic activity, florasulam, imazamox, malathion, non-target-site resistance mechanisms, target site resistance mechanism, tribenuron-methyl

## Abstract

Resistance to acetolactate synthase (ALS) inhibiting herbicides has recently been reported in *Glebionis coronaria* from wheat fields in northern Tunisia, where the weed is widespread. However, potential resistance mechanisms conferring resistance in these populations are unknown. The aim of this research was to study target-site resistance (TSR) and non-target-site resistance (NTSR) mechanisms present in two putative resistant (R) populations. Dose–response experiments, ALS enzyme activity assays, ALS gene sequencing, absorption and translocation experiments with radiolabeled herbicides, and metabolism experiments were carried out for this purpose. Whole plant trials confirmed high resistance levels to tribenuron and cross-resistance to florasulam and imazamox. ALS enzyme activity further confirmed cross-resistance to these three herbicides and also to bispyribac, but not to flucarbazone. Sequence analysis revealed the presence of amino acid substitutions in positions 197, 376, and 574 of the target enzyme. Among the NTSR mechanisms investigated, absorption or translocation did not contribute to resistance, while evidences of the presence of enhanced metabolism were provided. A pretreatment with the cytochrome P450 monooxygenase (P450) inhibitor malathion partially synergized with imazamox in post-emergence but not with tribenuron in dose–response experiments. Additionally, an imazamox hydroxyl metabolite was detected in both R populations in metabolism experiments, which disappeared with the pretreatment with malathion. This study confirms the evolution of cross-resistance to ALS inhibiting herbicides in *G. coronaria* from Tunisia through TSR and NTSR mechanisms. The presence of enhanced metabolism involving P450 is threatening the chemical management of this weed in Tunisian wheat fields, since it might confer cross-resistance to other sites of action.

## Introduction

*Glebionis coronaria* (L.) Cass. ex Spach, formerly called *Chrysanthemum coronarium* L., is one of the most troublesome broadleaf annual weeds in cereal crops in northern Tunisia. For years, Tunisian farmers faced challenges managing this weed in wheat fields. It has been estimated that wheat yield losses associated with high densities of *G. coronaria* is up to 75% (Hada et al., 2020). Typically, *G. coronaria* control is still heavily dependent on chemical treatments, mainly acetolactate synthase (ALS)-inhibiting herbicides and synthetic auxin herbicides (SAH). Recently, it has been proven that *G. coronaria* evolved resistance to sulfonylureas (SU), herbicides with potential cross-resistance to triazolopyrimidines – Type 1 (TP) family (Hada et al., 2020). Both herbicide families target the ALS enzyme, which catalyzes the first common step in the biosynthesis of leucine, isoleucine, and valine in plants (Duggleby et al., 2008; Liu et al., 2015). ALS is also the target of three other chemical families, namely, imidazolinones (IMI) (Shaner et al., 1984), sulfonanilides (BTP) (Stidham, 1991), and triazolinones (SCT) (Santel et al., 1999).

The ALS (also referred to as acetohydroxyacid synthase, AHAS) is the target site that is more resistance prone (Yu and Powles, 2014b). So far, evolved resistance to ALS inhibitors has been associated principally to target-site resistance (TSR), by one or more point mutations in the nuclear ALS gene, that disrupt herbicide binding and then reduce the sensitivity of the target enzyme to herbicides (Corbett and Tardif, 2006; Liu et al., 2015). Nowadays, 29 amino acid substitutions at eight different positions, namely, Al122, Pro197, Al205, Asp376, Arg377, Trp574, Ser653, and Gly654, have been documented (Tranel et al., 2019) in 165 weed species around the world (Heap, 2020). These amino acid substitutions result in various cross-resistance patterns among the five chemical families of ALS-inhibiting herbicides depending on weed species (Powles and Yu, 2010; Beckie and Tardif, 2012; Yu and Powles, 2014b; Tranel et al., 2019). For example, resistance to SU has been reported usually as a result of substitution in Pro197 position (Park et al., 2004) and in Gly654 position with less frequency. Amino acid substitutions in Al122, Ala205, Ser653, or Gly654 can endow resistance to IMI but not to SU family (Powles and Yu, 2010; Beckie et al., 2012). Both mutations in Trp574 and Asp376 positions resulted in wide cross-resistance to at least four families of ALS inhibitors (Tranel and Wright, 2002; Powles and Yu, 2010; Tranel et al., 2019).

Recent studies reported the occurrence of polygenic resistance mechanisms that reduce the amount of herbicide reaching ALS target site below lethal levels, known as non-target-site resistance (NTSR) (Deìlye, 2013). In NTSR, there are no significant changes at the ALS target enzyme or ALS enzyme expression level, although this subject is more complicated and less known in both biological and genetic contexts. Metabolic resistance or enhanced rates of herbicide metabolism is one of the major NTSR mechanisms in weeds. Often, it involves major enzymes such as cytochrome P450 monooxygenases (P450s) and glutathione S-transferases (GSTs) that confer to the resistant plant the ability to detoxify herbicides belonging to different chemical classes, and with different modes of action (MoA) (Preston, 2004; Busi et al., 2013, 2014; Yu and Powles, 2014a). ATP-binding cassette (ABC) transporters and enhanced activity of GSTs are also implicated in metabolic herbicide-resistant populations (Yu and Powles, 2014a). Enhanced metabolic resistance to ALS-inhibiting herbicides has been documented principally in grass weeds, such as *Alopecurus myosuroides* Huds. (Moss and Cussans, 1991), *Lolium rigidum* Gaudin (Yu et al., 2009), and *Echinochloa oryzoides* (*Ard.*) *Fritsch* (Yasuor et al., 2009). NTSR to ALS inhibitors have been rarely documented in dicot weeds. Examples are *Sinapis arvensis* L. (Veldhuis et al., 2000), *Amaranthus tuberculatus* (Moq.) Sauer (Guo et al., 2015), and *Papaver rhoeas* L. (Rey-Caballero et al., 2017).

Few cases have been found in which the accumulation of enhanced metabolism and punctual gene mutations coexist and increase resistance levels in weeds. The best-known well-studied case has been a population of *L. rigidum* resistant to chlorsulfuron in Australia (Christopher et al., 1991, 1992). *Rapistrum rugosum* (L.) All. is another weed showing high levels of resistance due to both target gene alteration and enhanced metabolism as resistance mechanisms (Hatami et al., 2016). Coexistence of TSR, such as point mutation, and NTSR (enhanced metabolism) in the same plant has been documented very rarely, particularly in dicots (Rey-Caballero et al., 2017).

This study aimed to investigate the potential TSR and NTSR mechanisms involved in *G. coronaria* resistance to ALS-inhibiting herbicides. The objectives were (1) to evaluate the resistant levels and the potential cross-resistance patterns based on *in vivo* and *in vitro* (ALS enzyme activity) dose–response experiments; (2) to determine if absorption, translocation, and/or enhanced metabolism endow resistance to *G. coronaria*-resistant populations; and (3) to determine the presence of possible point mutations in the ALS gene conferring TSR to *G. coronaria.*

## Materials and Methods

### Plant Materials

For all experiments, three populations of *G. coronaria* were used. Resistant (R) populations (R1 and R2) were selected from a previous screening of 10 putative R populations collected across the Bizerte region in northern Tunisia, with both populations showing highly resistance levels to SU herbicides (Hada et al., 2020). The susceptible (S) reference population, collected from the roadsides of the National Institute of Agronomy of Tunisia, had never previously received any herbicide treatments. Prior to every experiment, seeds were scarified using sand paper, soaked in 0.3% GA_3_ solution for 24 h at room temperature, and then germinated in a growth chamber at 27/17°C day/night and 16 h photoperiod under 350 μmol m^–2^ s^–1^ photosynthetic photon-flux density. Four days after, seedlings with two cotyledons just appearing were transplanted into pots filled with sand and peat mixture (1/3:2/3 *v/v*) and placed in a greenhouse under natural sunlight, 25/15°C, and ∼75% relative humidity, at the Universitat de Lleida and were watered as needed.

### Whole-Plant Dose–Response Experiment

The R (R1 and R2) and the S populations were sprayed at 2–4 leaf stage with tribenuron (Granstar 50 SX^®^, DuPont^TM^, 50 g kg^–1^), florasulam (Nikos supra^®^, Dow AgroSciences, 50 g L^–1^), and imazamox (Pulsar^®^ 40, BASF, 40 g L^–1^), at increasing doses (Table 1). A pretreatment with malathion (organophosphate insecticide, indicator of P450 enzymes involvement in resistance to ALS inhibitors by enhanced metabolism, Christopher et al., 1994) was applied to the plants 1 ½ h prior to both tribenuron and imazamox application. The dose of malathion applied was about 2000 g a.i. ha^–1^, which was the maximum dose without affecting *G. coronaria* survival or growth, according to preliminary trials (Supplementary Table 1). Either herbicides, malathion, or both were applied using a precision bench sprayer with two Hardi ISO LD-110-02 flat fan 110° opening nozzles, operating at a forward speed of 0.9 m s^–1^, 50 cm above plants, 200 L ha^–1^, and at a pressure of 215 kPa. Non-treated plants or plants with only malathion were used as controls. The experiment was arranged with six replicates (two plants per pot) per treatment (herbicide with malathion or herbicide without malathion), and per dose. After application, all pots were transferred to the greenhouse and arranged in a completely randomized design. Three weeks after treatment, the percentage of survivals and the fresh weights were recorded. The weight reduction was calculated in respect to corresponding untreated controls for each population and treatment. Experiments were performed twice.

**TABLE 1:** Herbicide range of doses applied to resistant (R) and susceptible (S) populations with or without malathion.

**Herbicide**	**Field rate (g a.i. ha^–1^)**	Malathion*	**Pop**	**Rates (g a.i. ha^–1^)**						
Tribenuron	18.7	±	R	18.75	37.5	56.25	75	150	300	0
		±	S	0.29	0.59	1.17	2.34	4.69	9.38	0
Florasulam	7.5	^–^	R	3.75	7.5	15	30	60	0	
		^–^	S	0.47	0.94	1.88	3.75	7.50	0	
Imazamox	50	±	R	25	50	75	100	200	0	
		±	S	1.56	3.13	6.25	12.5	25	50	

* (±) means that both treatments with malathion and without malathion were applied; (^–^) means that only herbicide without malathion treatment was applied.

### ALS Gene Sequencing

Plants from the S and the R1 and the R2 populations were subject to TSR investigation in a previous unpublished study. DNA was extracted from two susceptible plants from the S population, and four and eight tribenuron-resistant plants from R1 and R2 populations, respectively, using the rapid procedure described by Délye et al. (2015).

Primers GC-ALS 197 (5′-AGGTGGAGCTTCAATGGAGA-3′) and GC-ALS574 (5′-CCTGCAGGAATCATGGGTAA-3′) were used to amplify a 1300-bp ALS fragment of *G. coronaria* corresponding to 433 amino acids. PCR amplifications were performed using KOD FX (Toyobo, Osaka, Japan). Primers were used at a final concentration of 0.3 μM. The cycling program consisted of 94°C for 2 min followed by 35 cycles of 98°C for 10 s, 55°C for 15 s, and 72°C for 2 min. After amplification, PCR products were purified using the ExoSAP-IT PCR Product Clean-UP reagent prior to sequencing.

### Crude Enzyme Extraction and ALS Activity

Experiments were conducted to evaluate the ALS enzyme activity in the presence of ALS inhibiting herbicides following the method described by Rojano-Delgado et al. (2015). The ALS activity was measured by means of the quantification of acetoin production (nanomoles of acetoin per milligram of protein per hour). The acetoin product is formed by decarboxylation of acetolactate in the presence of acid. Acetoin reductions indicate that acetolactate production is inhibited due to less ALS enzyme activity during branched-chain amino acid synthesis (Dayan et al., 2015).

The activity of ALS enzyme was conducted in the presence of five ALS inhibitors from different chemical families (bispyribac, florasulam, flucarbazone, imazamox, and tribenuron) using crude extracts isolated from young foliar tissues of the R1, R2, and S populations. For enzyme extraction, 3 g of young leaf tissues were frozen and powdered using liquid nitrogen. Leaf powders were then homogenized in an extraction buffer (in a ratio of 1:3, tissue:buffer), containing 5 g of polyvinylpyrrolidone. The extraction buffer was prepared as a mixture of 0.1 M K-phosphate buffer solution (pH 7.5), 5 mM MgCl_2_, 10 mM sodium pyruvate, 100 μM flavin adenine dinucleotide, 50 mM thiamine pyrophosphate, 12 mM dithiothreitol, and glycerol–water (1:9, *v/v*). This homogenate was agitated for 10 min at 4°C, filtered through four layers of cheesecloth, and centrifuged for 20 min (20,000 rpm) to separate the supernatant. The supernatant obtained was immediately used for ALS enzyme activity assays.

Acetolactate synthase activity was assayed by adding 0.05 ml of enzyme extract to 0.1 ml of freshly prepared assay buffer [0.08 M potassium phosphate (KH_2_PO_4_/K_2_HPO_4_), pH 7.5, 0.15 M sodium pyruvate, 1.5 mM MgCl_2_, and 1000 μM FAD] and increasing concentrations of herbicides (0–1000 μM). After mixture and incubation (37°C for 1 h), the reaction was stopped by the addition of 0.05 ml of H_2_SO_4_ (3 M). The reaction tubes were then heated (15 min at 60°C) to facilitate decarboxylation of acetolactate to acetoin. Acetoin was detected as a colored complex (520 nm) formed after the addition of 0.25 ml of creatine (5 g L^–1^, freshly prepared in water) and 0.25 ml of α-naphthol (50 g L^–1^, freshly prepared in 5 M NaOH) and then incubated (60°C for 15 min). Background was determined using control vials in which the reaction was stopped before the incubation and subtracted. Maximum specific activity of ALS was measured in the absence of herbicides. The experiment was performed with three replications per herbicide concentration and per population, and repeated twice.

### Absorption and Translocation of ^14^C-Tribenuron Experiment

Radiolabeled herbicide solution was prepared by mixing commercial tribenuron herbicide at the recommended field dose (18.7 g a.i. ha^–1^), with labeled herbicide (^14^C-tribenuron, specific activity of 1.422 MBq mmol^–1^, Institute of Isotopes Co., Ltd., Budapest, Hungary). At the two-leaf stage, plants of the R and the S populations received on one of the two leaf surfaces four 0.5-μl droplets of herbicide mixture using a microapplicator (Hamilton PB 6000 dispenser, Hamilton, Co., Reno, NV, United States). Every plant received a total radioactivity of 0.67 KBq μl^–1^. Five plants per population and per sampling time (expressed as hours after treatment, HAT) were considered as repetitions. In each sampling time (12, 24, 48, and 72 HAT), plants were removed from pots, roots were carefully washed with distilled water, and treated leaf (TL) was separated from the rest of aerial section (AS) and root section (RS). Unabsorbed ^14^C-tribenuron was washed from every TL using 2 ml of acetone–water solution in a ratio of 1:1 (*v/v*), the washes were mixed with 15 ml of scintillation fluid (Ultima Gold^TM^, Perkin-Elmer, Packard Bioscience BV), and radioactivity was quantified by liquid scintillation spectrometry (LSS, 6000 TA scintillation counter, Beckman Instruments, CA, United States). The plant sections were dried for 48 h at 70°C, and combusted in a biological sample oxidizer (OX 500; R. J. Harvey Instrument, Tappan, NY, United States). The released ^14^CO_2_ was trapped in 18 ml of Oxysolve C400 (Zinsser Analytic, Frankfurt), and its associated radioactivity was determined by LSS.

The translocation of ^14^C-tribenuron in each plant section was determined and expressed as a percentage of total absorbed radioactivity. The average total recovery of the applied ^14^C-tribenuron was greater than 88%, both in S and R populations. For qualitative study of radiolabeled tribenuron, three plants per population were removed from the soil at 24, 48, and 72 HAT. Roots were rinsed and whole plants were pressed and dried at room temperature for 4 days. Then, plants were pressed against a phosphor storage film (25 cm × 2.5 cm, PerkinElmer Life and Analytical Sciences, Shelton, CT, United States), for 6 h. Dried plants were scanned using a phosphor imager Cyclone (Perkin-Elmer, Packard Bioscience BV).

The absorption and the translocation were calculated following these two expressions:

Absorption(%)

$$\;=\frac{\mathrm{the}\,\mathrm{radioactivity}\,\mathrm{recovered}\,\mathrm{from}\,\mathrm{plant}\,\mathrm{section}}{\mathrm{total}\,\mathrm{radioactivity}\,\mathrm{recovered}}\times 100$$

Translocation(%)

$$\;=\frac{\mathrm{the}\,\mathrm{absorbed}\,\mathrm{radioactivity}\,\mathrm{in}\,\mathrm{TL},\mathrm{AS},\mathrm{or}\,\mathrm{RS}}{\mathrm{absorbed}\,\mathrm{radioactivity}\,\mathrm{in}\,\mathrm{all}\,\mathrm{plant}\,\mathrm{sections}}\times 100$$

### Enhanced Metabolism Study of Imazamox Herbicide

Enhanced metabolism in *G. coronaria* populations was investigated by applying, at field rate, imazamox herbicide (50 g a.i. ha^–1^) alone and imazamox plus a pretreatment of malathion (2000 g a.i. ha^–1^). Two groups of controls were used: one group of non-treated plants and the second group of plants treated only with malathion. Plants were harvested at different times (0, 48, 72, and 96 HAT). Prior to the extraction, each plant was washed with 60 ml of water to remove traces of imazamox and soil on the leaf surface. The washed plants were divided into roots and shoots. The extraction and detection of metabolites were previously described by Rojano-Delgado et al. (2014). One-half gram of each plant sample was ground to powder in liquid nitrogen and mixed with a methanol–water solution (10 ml, 90:10 *v/v*), the mixture was ultra-sonicated during 10 min (at 70 W, duty cycle 0.7 s s^–1^) and then centrifuged for 15 min (at 20,000 rpm). Six milliliters of supernatant was collected and evaporated under an air stream, and later, 0.5 ml of extractant (methanol–water, 90:10 *v/v*) was added to reconstitute the sample. The new solution was filtered through a 45-μm pore-filter syringe (13 mm i.d. from Millipore, Carrigtwohill, Ireland) and used for liquid chromatographic quantification. Fifty microliters of the reconstituted solution was injected into a liquid chromatography system. A Gold HPLC (high-performance liquid chromatography) System (Beckman Coulter, Fullerton, CA, United States), equipped with a DAD (wavelength range 190–600 nm), was used to detect different analytes. The imazamox and metabolite separation was performed by using a hydrophilic interaction liquid chromatography column (HILIC; 3 μm particle size, 20 cm × 4.6 cm, Phenomenex, California, CA, United States), with a constant flow rate of 1.0 ml min^–1^ at 40°C. In this experiment, the wavelength of 240 nm was measured. In mobile phase A, 1% of acetic acid–water (*v/v*) was used, and in mobile phase B, pure methanol was used. The gradient elution program begun with first 5% mobile phase B and then linear gradient composed of (i) 5–20% methanol in 10 min; (ii) 20–80% methanol in 10 min; (iii) 80–100% methanol in 5 min; and (iv) 100–5% methanol in 10 min.

Target compounds were detected based on the retention times referring to the imazamox standard. The metabolites detected were quantified based on the calibration curve of imazamox, and results were given as concentrations (μg g^–1^) of imazamox and metabolites. The experiment was performed for each population using three replicates per sampling time, and two repetitions.

### Statistical Analysis

One-way ANOVA was performed using the SPSS-20 software (IBM, NY, United States) to analyze the dose–response data, for both whole-plant and ALS activity experiments, and means were compared using the Duncan *post hoc* pairwise test (*P*-value = 0.05). Four-parameter log-logistic regression models (Eq. 1) were fitted to determine the herbicide concentrations required to decrease the ALS activity (I_50_), fresh weight (ED_50_), and survival (LD_50_) by 50% in the three populations. The fitting for the dose–response experiment was performed using Sigmaplot 11.0 (Systat Software, San Jose, CA, United States), and the fitting for ALS enzyme activity was performed using R software (drc package). The resistance index (RI) of the R1 and the R2 populations to different ALS inhibitors tested were calculated as RI = LD_50_ (R)/LD_50_ (S), RI = ED_50_ (R)/ED_50_ (S), or RI = I_50_ (R)/I_50_ (S).

(1) $$y=c+\frac{\left(D-C\right)}{1+{\left(\frac{x}{x\,50}\right)}^{-b}}$$

*c*, the lower limit adjusted to 0; *d*, the upper limit adjusted to 100; *b*, Hill’s slope at *x*_50_ (inflection point, representing the effective herbicide dose required for 50% reduction in ALS activity, fresh weights, and survivals); *x* was the independent variable [dose of herbicide applied (g a.i. ha^–1^], and *y* was the dependent variable (ALS activity, fresh weight, or survival) expressed as a percentage of the untreated control.

For the ^14^C-tribenuron experiment, a two-way ANOVA was conducted to determine the effect of populations, sampling time, as well as their interaction on herbicide absorption and translocation. Data from the imazamox metabolism experiment was subjected to one-way ANOVA. Means were compared using Duncan *post hoc* pairwise test (*P*-value = 0.05).

## Results

### Cross-Resistance Pattern and Malathion Effect on Resistance

Both R1 and R2 populations exhibited resistance in response to tribenuron, imazamox, and florasulam based on survival and fresh weight reduction (%) recorded in this study. The highest resistance level (greater than 300) was attributed to tribenuron herbicide. In tribenuron-treated R1 and R2 plants, increasing rates of herbicide did not significantly affect the survival and fresh weight reduction (%), as the ED_50_ and LD_50_ values exceeded the highest applied dose (300 g a.i. ha^–1^). Thus, both R populations survived up to 16-fold the recommended field dose (Table 2).

**TABLE 2:** Parameters of the log-logistic equation of the dose–response regression curves of survival and fresh weight in *G. coronaria* S and R (R1 and R2) populations in presence and absence of malathion.

	**Herbicide**	**Malathion**	**Population**	**LD_50_/ED_50_ (g a.i./ha)^*a*^**	**Slope**	**RI**
*% Survivals*	Tribenuron	+	S	1.7	−1.7	–
			R1	>300	–	>300
			R2	>300	–	>300
		−	S	1.7	−2.4	–
			R1	>300	–	>300
			R2	>300	–	>300
	Imazamox	+	S	8.0	−1.8	–
			R1	49.5	−3.0	6.2*
			R2	49.9	−2.8	6.2**
		−	S	8.3	−3.0	–
			R1	63.8	−3.4	7.9**
			R2	69.7	−3.1	8.7*
	Florasulam	−	S	0.6	−0.9	–
			R1	5.2	−0.9	8.4***
			R2	10.2	−1.7	16.5***
*% Fresh weight*	Tribenuron	+	S	0.8	1.8	–
			R1	>300	–	>300
			R2	>300	–	>300
		−	S	1.4	2.7	–
			R1	>300	–	>300
			R2	>300	–	>300
	Imazamox	+	S	6.5	2.2	–
			R1	20.8	1.6	3.19***
			R2	24.8	1.7	3.81***
		−	S	5.6	2.4	–
			R1	27.7	1.6	4.25***
			R2	21.1	1.4	3.24***
	Florasulam	−	S	0.5	1.3	–
			R1	2.2	0.9	4.67***
			R2	4.0	1.1	8.66***

RI, resistance index. *P = 0.05, **P = 0.01, ***P = 0.001. ^*a*^LD_50_/ED_50_ represent the herbicide concentrations required to decrease by 50% the survival (%) and fresh weight (%) of *G. coronaria*, respectively.

**FIGURE 1:**
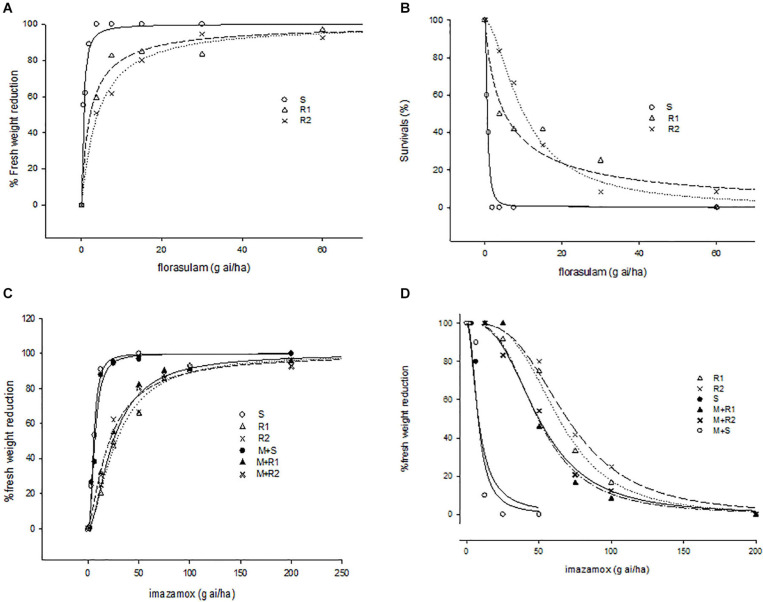
Response of fresh weight and survivals of susceptible and resistant populations of *G. coronaria* to different concentrations of florasulam **(A,B)** and imazamox **(C,D)**.

Regarding florasulam resistance, the R2 population was two times more resistant than the R1, showing higher survival rates and less fresh weight reduction (LD_50_ = 10.2 g a.i. ha^–1^ and ED_50_ = 4.0 g a.i. ha^–1^ respectively, Table 2 and Figure 1). Lower resistance levels were attributed to imazamox herbicides as presented by the RI (Table 2). The two R populations of *G. coronaria* showed similar survival rates with higher LD_50_ in R2 compared to the R1 plants (69.7 and 63.8 g a.i. ha^–1^, respectively). In contrast, fresh weight reduction was slightly higher in R1; ED_50_ was about 27.7 and 21.1 for the R1 and the R2, respectively.

A pretreatment with malathion partially increased the sensitivity of both *G. coronaria* R populations to imazamox (Figure 1 and Table 2). This was clearer in survival data, as both R1 and R2 exhibited 6.2 more resistance than the S plants, showing 21 and 29% reduction of resistance levels compared to plants treated only with imazamox. The results of fresh weight measurements supported these findings only for the R1 population (Table 2), together with the visual inspection of plant sizes (Supplementary Figure 1). Finally, malathion had no effect on tribenuron resistance *in G. coronaria.* These findings supported the hypothesis that enhanced imazamox metabolism may be present at least in the R1 population.

### ALS Activity

**FIGURE 2:**
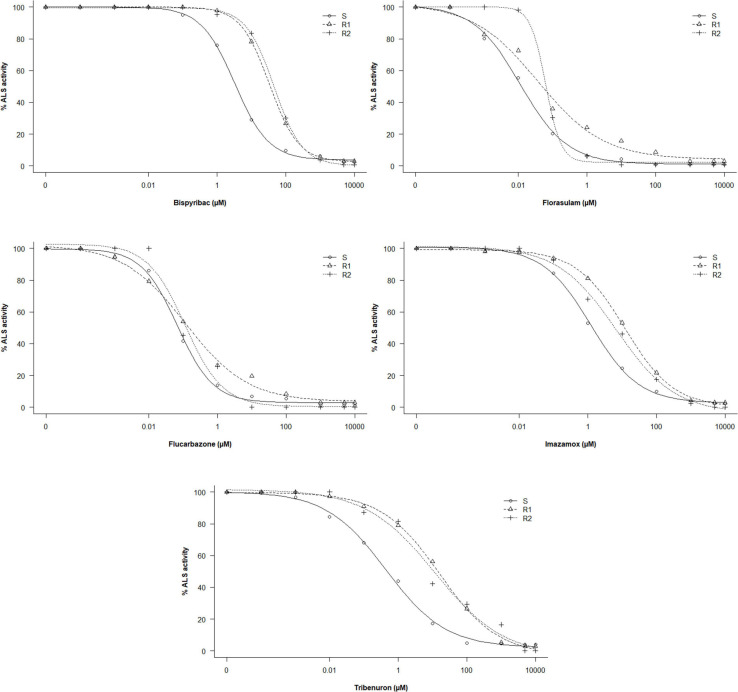
Dose–response curves of the ALS enzyme activity of *G. coronaria* populations exposed to increased concentrations (μM) of five herbicides.

In the absence of ALS inhibitors, the R1, R2, and S populations presented similar activity of extracted ALS. The basal activities were 305.9 (±18.6), 321.1 (±9.4), and 317.4 (±11.9) nmol acetoin mg TSP^–1^ h^–1^, respectively. However, results revealed significant differences in ALS activity between the R and the S populations when exposed to ALS-inhibiting herbicides (Table 3 and Figure 2). The I_50_ values ranged from 6.81 e^–02^ to 3.34 μM for the S population, 3.76 e^–02^ to 33.80 μM for R1, and 6.34 e^–02^ to 45.20 μM for R2.

**TABLE 3:** The resistance levels of different *G. coronaria* populations to bispyribac, florasulam, flucarbazone, imazamox, and tribenuron herbicides.

**Herbicides (chemical family)**	**Population**	**I_50_ (μM)**	***F-*value**	**RI**
Bispyribac (BTP)	S	3.34		–
	R1	33.80	48.945***	10.242
	R2	45.20		13.532
Florasulam (TP)	S	1.15 e^–02^		–
	R1	3.76 e^–02^	11.732***	3.270
	R2	6.34 e^–02^		5.513
Flucarbazone (SCT)	S	6.81 e^–02^		–
	R1	1.17 e^–01^	11.645***	1.718
	R2	1.15 e^–01^		1.689
Imazamox (IMI)	S	1.22		–
	R1	12.03	4.6486***	9.861
	R2	6.68		5.475
Tribenuron (SU)	S	0.44		–
	R1	16.02	2.2309***	36.409
	R2	11.91		27.068

SU, sulfonylurea; IMI, imidazolinone; TP, triazolopyrimidine; PTB, pyrimidinyl-thiobenzoates; SCT, sulfonyl-aminocarbonyl-triazolinone; I_50_, herbicide dose required to inhibit ALS activity by 50% compared with that of the untreated control, R1 and R2, resistant population; S, susceptible population. RI, resistance index = I_50_ (R1)/I_50_ (S); (RI) = I_50_ (R2)/I_50_. ***: the differences between populations are highly significant according to the Duncan test (*P* = 0.001).

Higher values of RI in tribenuron and bispyribac were found. Resistant plants of the R1 and the R2 required a tribenuron concentration of 36 and 27 times higher respectively than the S plants for similar inhibition of ALS activity. The I_50_ values were only 1.7-fold higher in both R populations compared with the S population treated with flucarbazone, indicating almost no resistance to this herbicide. Results also showed that resistance to tested herbicides varied among the two populations R1 and R2. The quantification of the ALS activity revealed that the R1 was more resistant to tribenuron and imazamox, while the R2 was more resistant to bispyribac and florasulam. Based on the RI estimated by the ALS activity assays, resistance to ALS inhibitors may be classified as follows: flucarbazone < florasulam < imazamox < bispyribac < tribenuron. Furthermore, our results might suggest that both R1 and R2 populations developed cross-resistance to four out of the five chemical families of the ALS-inhibiting herbicides used in this study.

### ALS Sequencing

Comparison of ALS gene sequences in susceptible (accession number MW598184) and resistant plants (accession numbers MW598185 to MW598189) revealed three non-synonymous mutations at positions 197, 376, and 574 standardized to the ALS protein sequence of *Arabidopsis thaliana* (Supplementary Figure 2), which are already known to be involved in sensitivity to ALS-inhibiting herbicides in weeds (Tranel et al., 2019). At codon 197, the amino acid substitution Pro (CCA) to Thr (ACA) was detected in susceptible and resistant plants. However, the Arg197, Ser197, and Gln197 substitutions caused by variable nucleotide positions were only detected in the R2 population. The amino acid change at position 376 was caused by a substitution from Asp (GAT) to Glu (GAA) in three plants from the R2 population. A point mutation at the second base of the amino acid Trp (TGG), which resulted in the substitution by Leu (TTG), was found in one homozygous and one heterozygous plant from the R1 population (Table 4). These findings showed that most R plants sequenced contained at least one mutant-resistant allele, suggesting that the TSR mechanism is relevant in *G. coronaria* resistance to ALS-inhibiting herbicides.

**TABLE 4:** Different amino acid substitutions detected at codons Pro197, Asp376, and Trp574 of the ALS gene from S (susceptible) and R1 and R2 populations (resistant) of *G. coronaria* based on amino acid positions in *Arabidopsis thaliana.*

**Population**	**Plant**	Substitutions*		
		**Pro197 CCA**	Asp376 GAT	Trp574 TGG
S	1	Pro/Thr CCA**/A**CA*	–	–
	2	Pro/Thr CCA**/A**CA	–	–
R1	1	–	–	Leu/Leu T**T**G/T**T**G
	2	–	–	Trp/Leu TGG/T**T**G
	3	Thr/Thr **ACA/A**CA	–	–
	4	Pro/Gln **A**CA**/CA**A	–	–
R2	1	Pro/Ser CCA**/T**CA	–	–
	2	Thr/Ser **A**CA**/T**CA	–	–
	3	Gln/Gln C**A**A**/**C**A**A	–	–
	4	Gln/Gln C**AA/**C**A**A	–	–
	5	Pro/Arg CCA**/**C**G**A	Asp/Glu GAT/GA**A**	–
	6	–	Asp/Glu GAT/GA**A**	–
	7	–	Asp/Glu GAT/GA**A**	–
	8	–	Asp/Glu GAT/GA**A**	–

*Nucleotide changes are in bold.

### Absorption and Translocation

Statistical analysis showed no significant differences (*P* > 0.05) in absorption patterns between populations, as the penetration of labeled herbicide remained asymptotic. Overall, the ^14^C−tribenuron absorption was less than 30% for all populations. No significant differences were found in absorption percentages between R and S populations over time, although the R2 population was able to absorb more labeled herbicide (27%) at the end of the experiment, compared to the R1 and the S populations (22 and 24%) (Table 5).

**TABLE 5:** Absorption (expressed as % of recovered radioactivity) and translocation to different plant organs (expressed as % of absorbed radioactivity) of ^14^C-tribenuron in S, R1, and R2 populations of *G. coronaria* at 12, 24, 48, and 72 HAT.

**Population**	**HAT**	**Absorption (%)**	**Translocation (%)**		
			**Treated leaf (TL)**	**Rest of aerial section (AS)**	**Root section (RS)**
S	12	24.0 ± 4.1	92.2 ± 1.9	7.3 ± 1.9	0.5 ± 0.1
	24	29.6 ± 9.6	96.1 ± 0.3	3.5 ± 0.3	0.4 ± 0.2
	48	23.2 ± 3.6	89.7 ± 2.6	9.9 ± 2.6	0.4 ± 0.1
	72	23.8 ± 1.4	91.4 ± 2.3	8.2 ± 2.3	0.4 ± 0.0
R1	12	20.6 ± 1.5	93.6 ± 2.3	5.8 ± 2.2	0.5 ± 0.0
	24	22.9 ± 2.0	90.9 ± 3.2	8.6 ± 3.2	0.4 ± 0.0
	48	22.9 ± 3.6	92.6 ± 2.0	7.1 ± 2.0	0.4 ± 0.0
	72	21.8 ± 1.5	93.2 ± 2.4	6.4 ± 2.4	0.4 ± 0.0
R2	12	22.0 ± 2.9	86.5 ± 1.3	13.0 ± 1.2	0.5 ± 0.1
	24	21.0 ± 1.9	88.2 ± 1.4	11.1 ± 1.5	0.7 ± 0.3
	48	22.0 ± 3.4	90.5 ± 0.4	9.0 ± 0.5	0.5 ± 0.1
	72	27.4 ± 2.5	90.9 ± 2.1	8.8 ± 2.0	0.3 ± 0.0

Means of five repetitions per biotype ± values of standard errors are given.					

**ANOVA**		**Absorption (%)**	**Treated leaf (TL)**	**Rest of aerial section (AS)**	**Root section (RS)**

Population		*P* = 0.331^*ns*^	*P* = 0.016**	*P* = 0.019**	*P* = 0.459^*ns*^
HAT		*P* = 0.712^*ns*^	*P* = 0.856^*ns*^	*P* = 0.873 ns	*P* = 0.258^*ns*^
Population × HAT		*P* = 0.542^*ns*^	*P* = 0.134^*ns*^	*P* = 0.142 ns	*P* = 0.497^*ns*^

^*ns*^No significant differences were found; **significant differences were found at *P* = 0.05 based on Duncan test.

According to the translocation data (Table 5), the highest amount of the labeled herbicide remained in the treated leaves, representing more than 90% of the recovered radioactivity at 72 HAT. The lowest amounts of radioactivity were detected in both shoots and roots in a similar distribution pattern for all populations. However, significant differences in herbicide translocation were found between populations until 24 HAT. Only 86% of radioactivity remained in the R2-treated leaves compared to 92 and 93% in the S and the R1 plants, respectively. Similarly, 13% of radioactivity was found in the aerial part of the R2 plants, while only 7 and 5% of radioactivity were detected in the S and the R1, respectively. These differences remained until 24 HAT but faded over time, indicating a similar pattern of tribenuron herbicide translocation in all tested populations.

**FIGURE 3:**
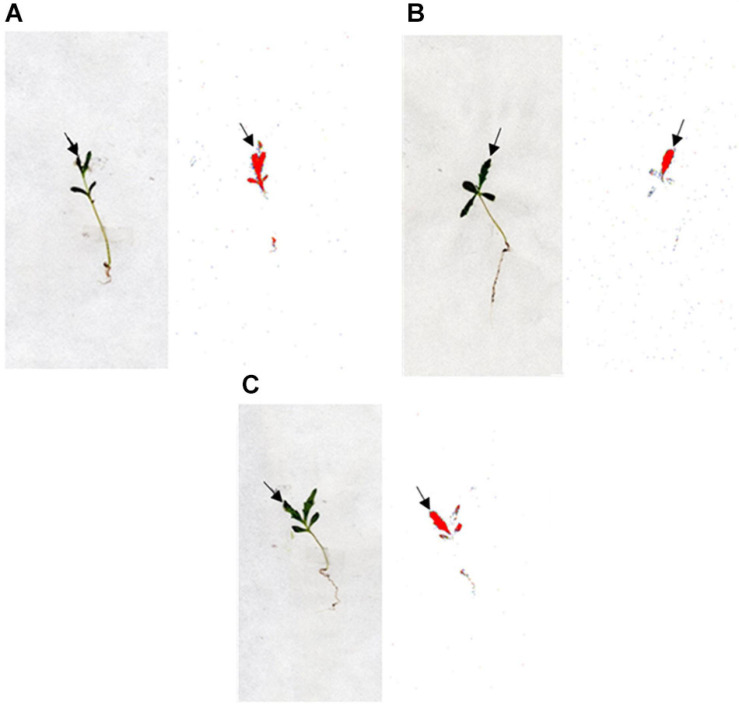
Digital photographs (plants on the left) and autoradiographic photographs of the phosphor imager (plants on the right) showing the distribution of ^14^C-tribenuron within *G. coronaria* plants of population S, R1, and R2 (**A**, **B**, and **C**, respectively) at 72 HAT. The highest concentration of ^14^C is highlighted in red.

Thus, neither absorption nor translocation mechanisms could be responsible for the high levels of tribenuron resistance observed in both R1 and R2 populations. Phosphor images illustrated a similar level of ^14^C−tribenuron movement in the R and the S populations, showing minimal translocation to the rest of the AS and the roots (Figure 3).

### Imazamox Metabolism Studies

In all sampling times, higher amounts of herbicide were detected in the whole plants (foliar and roots) of the S population, especially at 72 and 96 HAT, compared to the R1- and the R2-resistant populations (Table 6). In the absence of malathion, the amount of imazamox was significantly different among populations. However, it was statistically similar in leaves when plants were pretreated with malathion. Chromatographic analysis permitted the detection of a hydroxyl metabolite of imazamox (imazamox-OH) at 20 min (Supplementary Figure 2). No imazamox metabolite was formed in the leaves and roots of plants pretreated with malathion in all tested populations.

**TABLE 6:** Imazamox concentration and its hydroxyl metabolite (Imaza-OH) in foliar and root extracts from R (R1 and R2) and S populations of *G. coronaria* at 48, 72, and 96 HAT (μg/g^−1^).

**HAT**	**Pop**	**^–^Malathion**				**+ Malathion**			
		**Imazamox**		**Imaza-OH**		**Imazamox**		**Imaza-OH**	
		**Foliar**	**Root**	**Foliar**	**Root**	**Foliar**	**Root**	**Foliar**	**Root**
48	S	32.4 ± 2.1b	9.9 ± 0.4b	ND	ND	30.2 ± 2.1a	12.7 ± 19b	ND	ND
	R1	25.2 ± 1.9a	1.7 ± 0.4a	5.9 ± 0.2	ND	29.6 ± 0.5a	9.7 ± 0.4ab	ND	ND
	R2	33.0 ± 2.4b	ND	ND	ND	29.2 ± 0.9a	7.0 ± 1.3a	ND	ND
	*F*-value	8.07*	132.5***	–	–	0.3^*ns*^	8.8*	–	–
72	S	36.0 ± 2.8a	25.5 ± 2.7c	ND	ND	39.3 ± 0.8a	31.9 ± 3.1b	ND	ND
	R1	48.1 ± 2.2b	2.9 ± 0.5b	7.9 ± 0.4	2.8 ± 0.5	38.0 ± 0.6a	26.9 ± 0.5b	ND	ND
	R2	48.2 ± 1.6b	2.7 ± 0.3a	ND	ND	36.4 ± 2.4a	15.7 ± 2.1a	ND	ND
	*F*-value	19.6**	112***	–	–	1.9^*ns*^	29.3***	–	–
96	S	31.3 ± 3.9a	42.5 ± 1.7c	ND	ND	38.5 ± 1.5a	50.0 ± 1.3c	ND	ND
	R1	42.5 ± 4.0b	8.8 ± 0.b	8.9 ± 0.3b	9.9 ± 1.6	36.6 ± 1.1a	38.9 ± 0.2b	ND	ND
	R2	63.3 ± 3.0c	4.0 ± 0.3a	1.6 ± 0.4a	ND	36.9 ± 1.1a	32.0 ± 1.2a	ND	ND
	*F*-value	43.2***	858.4***	732.1***	–	1.4^*ns*^	181.1***	–	–

*P = 0.05, **P = 0.01, ***P = 0.001 ^*a*,*b*,*c*^Different letters denote significantly differences between populations in imazamox concentration and its hydroxyl metabolite (Imaza-OH) in foliar and root extracts at different sampling time according to the Duncan test (*p* = 0.05).

In the absence of malathion pretreatment, the imazamox-OH was detected at all sampling times for the R1 population and only at 96 HAT for the R2 population. Additionally, imazamox metabolites were not detected in the S plants over time (Supplementary Figure 3), indicating different metabolism patterns between the R and the S populations. These results may suggest the important role played by a P450 enzyme system, inhibited by the pretreatment with malathion, conferring greater resistance through the hydroxylation of imazamox (first step in metabolism) in the R1 and the R2 plants when exposed to the herbicide. Statistical analysis showed significant differences in imazamox and imazamox-OH concentrations between the R1 and R2 populations. At 48 HAT and in the absence of malathion, the amount of imazamox in the R1 population was significantly lower than in the R2. Imazamox herbicide was detected in the roots of the R1 population while it was found only in foliar part of the R2. The imazamox-OH was first detected in leaves of the R1 population and then in both leaves and roots at the concentrations of 7.9 μg g^–1^ and 2.8 μg g^–1^, respectively. No metabolite was detected in the R2 population until 96 HAT. The detection of imazamox-OH in the R2 population occurred at a very low concentration (1.6 μg g^–1^), compared to the one detected in the R1 (8.9 μg g^–1^).

## Discussion

Results from this study revealed the development of cross-resistance to ALS-inhibiting herbicides in two *G. coronaria* populations from the Bizerte region in northern Tunisia. Since their introduction in 1999, the SU herbicides were excessively used by the Tunisian wheat growers to control troublesome grass weeds such as *L. rigidum* (Khammassi et al., 2020) and dicotyledonous weeds. The main reason was their broad-spectrum weed control at low rates. A previous survey, conducted in the same region, showed that florasulam was frequently used in a mixture with SAH to control *G. coronaria* in cereal crops (Hada et al., 2020) while imazamox has never been applied on the weed to our knowledge.

Data on the survival and the fresh weight reduction showed that both R1 and R2 populations exhibited high resistance levels to tribenuron and were less resistant to florasulam and imazamox. Similar results were found in *P. rhoeas* showing that the degree of resistance varied among ALS inhibitors, with RI lowest for florasulam and imazamox than for tribenuron (Kaloumenos et al., 2011). Our findings are in line with the only known study conducted by Tal and Rubin (2004) on the occurrence of resistance to ALS-inhibiting herbicides in *G. coronaria* populations, showing high RI (greater than 729) to tribenuron and cross-resistance to all chemical families of ALS-inhibiting herbicides (RI ranging between 4 and 48). There is an abundant literature demonstrating resistance to ALS-inhibiting herbicides in dicotyledonous weed species including *Salix alba* in Spain (Rosario et al., 2011), *Myosoton aquaticum* (L.) Moench in China (Liu et al., 2015), *R. rugosum* in Iran (Hatami et al., 2016), *P. rhoeas* in Spain (Rey-Caballero et al., 2017), and *Amaranthus palmeri* in the United States (Küpper et al., 2017). It is common for these species to develop resistance to herbicides belonging to SU chemical family, as well as cross-resistance to herbicides from other families within ALS inhibitors.

The results of *in vitro* ALS activity further confirmed the cross-resistance to florasulam, imazamox, tribenuron, and also to bispyribac. A lower RI (<2) was determined for flucarbazone. Gene sequencing revealed amino acid replacements in positions Pro197, Asp376, and Trp574. Both mutations in Trp574 and Asp376 positions resulted in wide cross-resistance to at least four families of ALS inhibitors (Tranel and Wright, 2002; Powles and Yu, 2010; Tranel et al., 2019). Amino acid substitutions in Pro197 were previously reported in resistant *G. coronaria* (Tal and Rubin, 2004). These authors found that Thr197 and Ser197 substitutions conferred high resistance levels to tribenuron. However, in this study, the Thr197 substitution was also detected in susceptible plants. In *Monochoria korsakowii*, a Pro197Leu codon known to confer resistance in other species was detected in a pseudogene (Iwakami et al., 2020). These point mutations are widely reported in other dicotyledonous weeds as endowing resistance to SU (Cruz-Hipolito et al., 2013; Yu and Powles, 2014a; Liu et al., 2015) and cross-resistance to other chemical families (Hatami et al., 2016). Our findings, which are in line with the previous studies, suggest that the cross-resistance pattern found in this study may be explained by the substitutions in Pro197, Asp376, and Trp574 and that target-site alteration may be the dominant mechanism of resistance developed by *G. coronaria* to ALS-inhibiting herbicides. However, it is important to determine the copy number of ALS genes and their transcription in *G. coronaria* to better understand the evolution of target-site herbicide resistance. Despite the fact that SU and TP herbicide families are the only ones applied by Tunisian farmers in wheat fields, the weed has developed resistance to other ALS inhibitor families that have never been used before, which might hamper the use of any new ALS inhibitor in the cereal fields in this northern region of Tunisia.

The levels of tribenuron absorption and translocation in the R and the S populations were also investigated in this study. Based on the results, neither absorption nor translocation contributed as mechanisms to *G. coronaria* resistance to ALS-inhibiting herbicides. Indeed, both mechanisms rarely underlay resistance to the ALS inhibitors (Veldhuis et al., 2000; Cruz-Hipolito et al., 2013; Riar et al., 2013; Yu and Powles, 2014b). These results further support the reports from other studies on *Conyza sumatrensis* (Retz.) E. Walker (Osuna and De Prado, 2003), *S. alba* (Rosario et al., 2011), or *R. rugosum* (Hatami et al., 2016). However, several resistant weed species such as *P. rhoeas* (Rey-Caballero et al., 2017) were able to translocate more herbicide than the susceptible ones, suggesting that ALS inhibitors may affect the transport of assimilates into the phloem. Previous work on *Pisum sativum* L. reported that carbohydrates were excessively accumulated in leaves of S plants after herbicide application (Zabalza et al., 2004). Similar results were also reported on *Thlaspi arvense* L. (Bestman et al., 1990).

In dose–response experiments, pretreatment with the P450 inhibitor malathion had no effect on the efficacy of tribenuron. However, it partially synergized with imazamox, leading to a shift toward sensitivity in both R populations. This indicates that P450 would be involved in the resistance response of *G. coronaria* to ALS-inhibiting herbicides, at least those of the IMI family. The P450 superfamily is the largest enzymatic protein family in plants, linked to vital functions, participating in the synthesis of fatty acids, sterols, and hormones. Members of this superfamily are involved in multiple metabolic pathways with distinct and complex functions, mediating a vast array of reactions (Jun et al., 2015). Furthermore, it is known that this family of enzymes is responsible for the detoxification processes of xenobiotics, in particular of herbicides in plants (Powles and Yu, 2010). Malathion has an inhibiting effect on P450 in plants, which can no longer catalyze herbicide degradation. Therefore, R plants may totally or partially, as in the case of this study, lose their resistance.

Enhanced metabolism, likely meditated by P450 in both R1 and R2 populations, was confirmed by HPLC experiments. This is the first report of the presence of NTSR mechanisms in *G. coronaria*. Indeed, imazamox-hydroxyl metabolite was detected in both R1 and R2 populations and disappeared with the pretreatment with malathion. Comparing both R populations, the R1 was able to detoxify imazamox faster than the R2 did. In fact, the faster an herbicide is metabolized, the less it is available for translocation and activity at the site of action (Zimdahl, 2007). This may explain the differences in the translocated amounts of imazamox between the two R populations. Previous works in *L. rigidum* (Busi et al., 2011) and *A. myosuroides* (Petit et al., 2011) pointed out that metabolic resistance can be controlled by polygenic loci and that these loci were involved in NTSR to ALS inhibitors depending on plants and populations. Recent studies characterized P450-based metabolic cross-resistant individuals in *L. rigidum* and hypothesized that the genetic control was probably of polygenic and quantitative nature at the population level (Yu and Powles, 2014b; Han et al., 2021). Considering the complexity of metabolic herbicide resistance and the diversity and number of P450s in plants, we hypothesize that the differences observed in metabolism speed and metabolite amounts between the R1 and the R2 populations could be explained by herbicide and environmental selection pressures, particularly the history of herbicide applications in the fields where R populations were collected (Yu and Powles, 2014b; Hada et al., 2020). Owing to the genetic diversity of the weedy species, differences in the P450 activity and presence of other genes may be involved in the resistance of *G. coronaria* to imazamox, which can be observed even in different individuals of a single population as reported by Yu and Powles (2014b). This ability of P450 to metabolize existing and yet-to-be-discovered herbicides is a serious threat to herbicide sustainability. It is unpredictable and could confer resistance to *G. coronaria* regardless of the herbicide molecule or MoA. This may include SAH, which are very effective in managing this newly resistant weed as reported recently (Hada et al., 2020). It has already been suggested that a common P450 can metabolize SAH and imazamox in multiple herbicide-resistant *P. rhoeas* (Torra et al., 2021).

Even no evidence was found on the presence of enhanced metabolism to tribenuron. Both R populations were able to metabolize, in part, imazamox, which belongs to the same MoA. It suggests that only one unit from the P450 system might contribute to the imazamox detoxification. This raises the hypothesis that enhanced metabolism is present in *G. coronaria* populations as a mechanism of resistance to herbicides. However, it could be hidden under the high resistance conferred by point mutations in the target gene. This observation is similar to the aforementioned reports on *P. rhoeas* (Rey-Caballero et al., 2017) and *R. rugosum* (Hatami et al., 2016), with multiple resistance mechanisms.

## Conclusion

In this study, both point mutations in ALS gene and enhanced metabolism were confirmed as mechanisms endowing resistance to ALS-inhibiting herbicides in *G. coronaria*, a weed spreading in the most important cereal land in Tunisia. Amino acid substitutions could explain the high resistance levels for tribenuron herbicide and cross-resistance to imazamox, florasulam, and bispyribac. Meanwhile, enhanced metabolism was only detected for imazamox herbicide in both tested R populations, showing that *G. coronaria* weed is able to detoxify herbicides never received before. Therefore, the management of these weed R populations with NTSR mechanisms is challenged as reported by previous researches (De Prado and Franco, 2004; Yu et al., 2009; Yu and Powles, 2014a). So far, the relative importance of enhanced metabolism in the resistance response of *G. coronaria* to ALS inhibitors in field conditions is unknown. Furthermore, we ignore the interaction between TSR and enhanced metabolism, at both physiological and genetic levels. This should trigger more research, exploring the multiple resistance factors by means of transcriptome analyses and inheritance studies to understand the potential risk of the multiple resistance mechanisms evolving in *G. coronaria* in wheat fields.

## Data Availability Statement

The original contributions presented in the study are included in the article/Supplementary Materials, further inquiries can be directed to the corresponding author/s.

## Author Contributions

AR-D performed metabolism experiment and ALS activity assay, while JM conducted absorption/translocation assay. ZH and JT performed dose–response experiment, participated in absorption/translocation experiment, and drafted the manuscript. CP-B elaborated the Translocation Phosphor image. YM performed ALS gene extraction and amplification, and worked on sequencing results. All authors reviewed and contributed to the manuscript.

## Conflict of Interest

The authors declare that the research was conducted in the absence of any commercial or financial relationships that could be construed as a potential conflict of interest.
