# Supplementary material for: Point Mutations as Main Resistance Mechanism Together With P450-Based Metabolism Confer Broad Resistance to Different ALS-Inhibiting Herbicides in Glebionis coronaria From Tunisia
Source: Front Plant Sci. 2021 Apr 1;12:626702. doi: 10.3389/fpls.2021.626702 (PMC8047477; doi:10.3389/fpls.2021.626702)
Supplement: Supplementary file 1 [file Data_Sheet_1.docx]

**Supplementary Data**

**Tables**

Supplementary Table 1. Fresh weight (g pot^-1^) of three *Glebioniscoronaria* populations, one susceptible (S) and two resistant (R1 and R2) to ALS inhibiting herbicides. Plants were untreated (Control), pre-treated only with malathion at two doses, 1000 g a.i. ha^-1^ (M1000) or 2000 g a.i. ha^-1^ (M2000), only with imazamox at 60 g a.i. ha^-1^ (IMI), or malathion treatments followed by an imazamox application (IMI+M1000 and IMI+M2000) treatment at 60 g a.i. ha^-1^). Two plants were established in each pot, with four replicates (pot) per treatment.

| Population | Control | M1000 | M2000 | IMI | IMI+M1000 | | IMI+M2000 | |
| --- | --- | --- | --- | --- | --- | --- | --- | --- |
| S | 1.22±0.40 | 1.29±0.07 | 1.04±0.48 | 0.00±0.00 | | 0.00±0.00 | | 0.03±0.00 |
| R1 | 1.56±0.36 | * | 1.35±0.35 | 0.53±0.46 | | * | | 0.31±0.32 |
| R2 | 1.70±0.99 | 1.92±0.20 | 1.33±0.33 | 0.57±0.35 | | 0.5±0.02 | | 0.31±0.29 |

* no plants were available for these treatments.

**Figures**


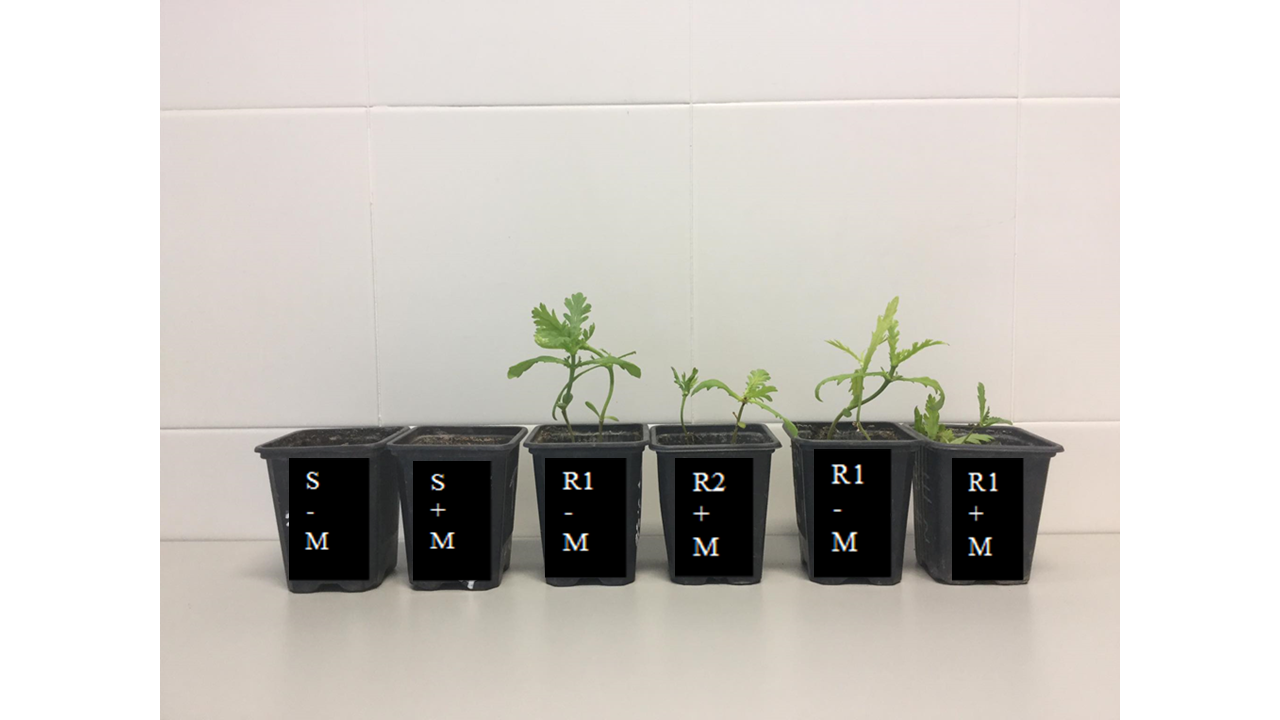


Supplementary Figure 1. Photograph of *Glebioniscoronaria* plants from three populations, one susceptible (S), and two resistant (R1 and R2) to ALS inhibitors, treated with imazamox (50 g a.i. ha^-1^), without (- M), or with a pre-treatment with the P450 inhibitor malathion (2000 g a.i. ha^-1^).


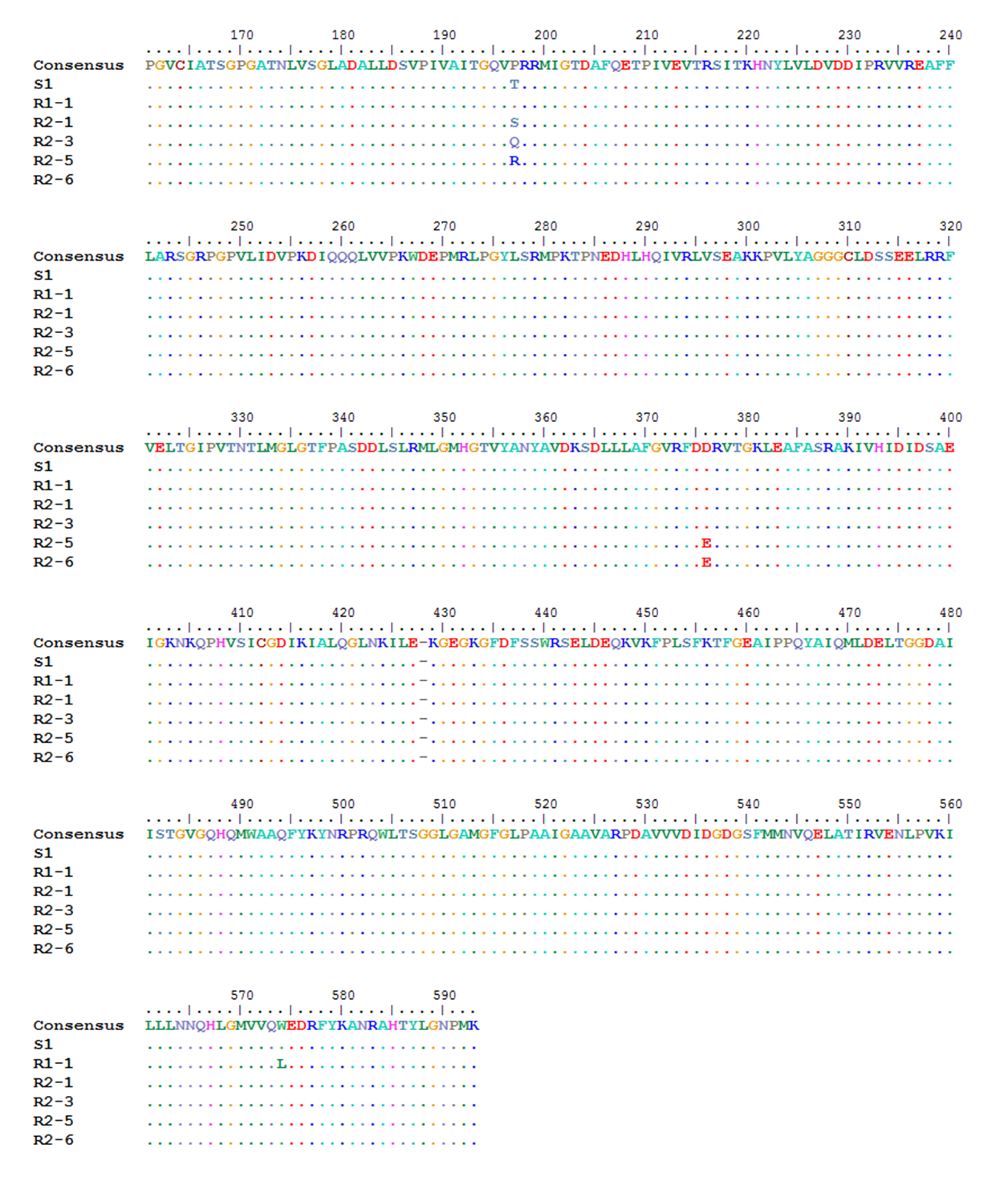


Supplementary Figure 2. Different ALS protein haplotypes detected in the S (susceptible), R1 and R2 populations (resistant). Dots indicate amino acids identical to those found in the consensus sequence.


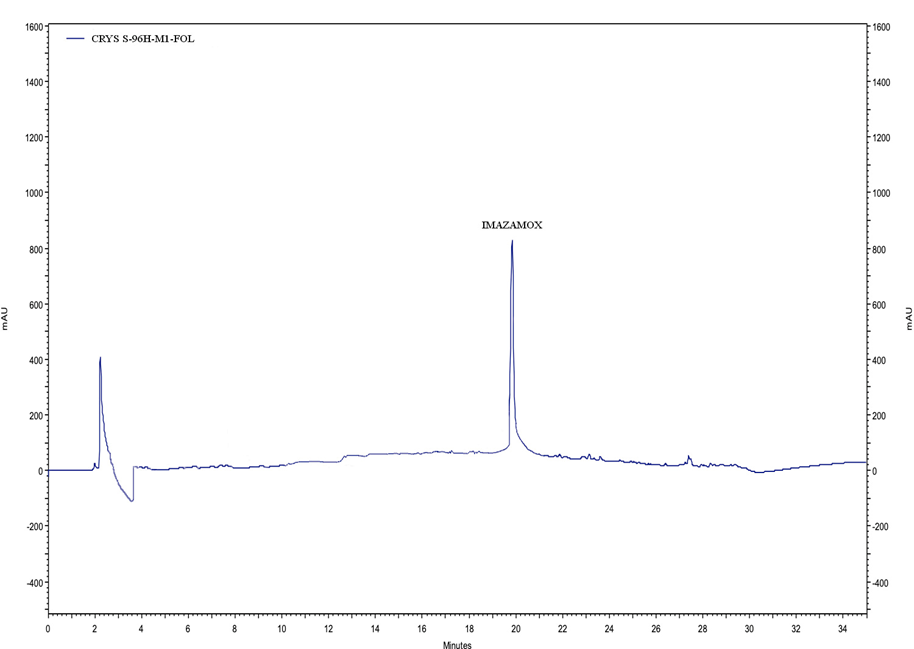


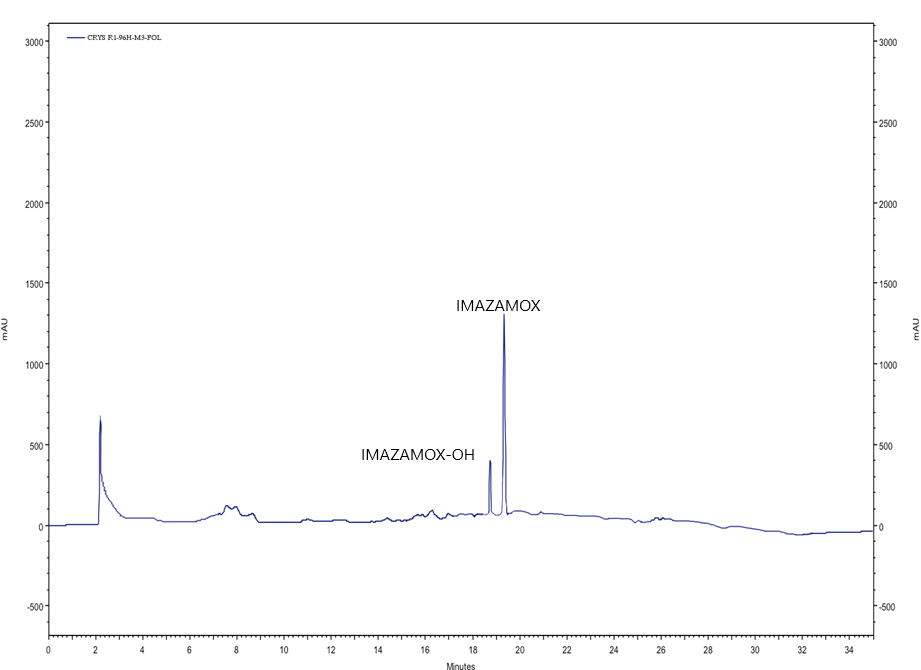


Supplementary Figure 3. HPLC chromatograms of one susceptible (above) and one resistant plant (below) to ALS inhibitors of *Glebioniscoronoria* from Tunisia at 96 HAT. The presence of the parental herbicide imazamox (both cases) or the hydroxyl metabolite (imazamox-OH) for the resistant plant (R1population) is indicated.
